# British Hospital and Ambulance Work in France

**Published:** 1916-10-07

**Authors:** 


### BRITISH HOSPITAL AND AMBULANCE WORK IN FRANCE.

### Some Impressions of a Visit.

By A LAY HOSPITAL OFFICER.

It was recently suggested, quite seriously, by a lieutenant-colonel of the R.A.M.C., that instead of the first-aid posts in the trenches, or in addition to them, there should be a series of dug-out hospitals where gravely wounded cases could remain for at least some days. "One hundred feet deep" was the idea of measurement, but the R.E.s would probably have something to say to that. To one who has seen something of the adaptations and expedients so cleverly brought into use by those responsible for the British Hospital Service in France the suggestion is not so startling as it may appear on first examination; solution of difficulties in respect of ventilation and sanitation is with the R.A.M.C. such an everyday concern as hardly to weigh in forming an opinion. Moreover, we have learned so much from the enemy of the possibilities of a hole in the ground. The chief obstacle would be want of mobility, for after two years' occupancy of practically the same lines and in view of recent liveliness, the dug-out hospital would soon be left in the rear, with the object of its formation defeated. So, unless we settle down to another prolonged period of stagnation elsewhere, all the wounded will follow the same road as heretofore: from the first-aid post to the advanced dressing station, thence to the main dressing station, and on to the clearing station (near that mysterious locality known as "railhead"), where the very dangerously wounded will be kept for a space and the others sent by train to the base hospitals. Then, a little later, according to their condition, the men will go either to a convalescent camp, preparatory to return to duty, or by the Red Cross steamer to England. It seems a long road with many halting-places, yet a wounded man can travel, and has travelled, from the first-aid post to a hospital in London within fifteen hours.

### The Magic Word "Evacuation."

After due regard has been paid to the wounded who would suffer from undue transport the chief motto of the service is "Movement," or, to use the service word, "Evacuation," and the average stay at a base hospital, where most remain the longest, is under six days. Sometimes, when the trains are arriving frequently, the Entraining Medical Officer and his men do not take their clothes off for several days together.

The "E.M.O." is a busy man: when he receives advice from railhead as to how many trains are on the way, and how many cases they contain, the number divided into the two groups "sitting" and "lying," he must consult his lists of vacant beds, and decide to which hospitals in his district the cases must be allocated, taking care that an undue proportion of heavy cases are not sent to one place. He then instructs the hospitals as to the nature of the take-in, and arranges for the requisite number of bearers and ambulances. He is the responsible vehicle for confidential documents passing between the clearing station and a base hospital containing information of a special nature in respect of cases; in addition, he is the custodian of mislaid property of the wounded and the effects of unconscious patients.

### A Moving Hospital.

The trains are on their way. One may be a proper khaki train specially built for its business, another an ordinary English or French passenger train adapted to Red Cross work. Either will carry its staff of three medical officers, three sisters, and a sufficient company of orderlies; these all live on the train, and are provided with sleeping accommodation, a kitchen, and compartments for taking meals. There is also a kitchen for the patients, an operation room, and other necessary offices. The whole is a model of economy in space. Invariably the driver of the train is French, and travel is at a uniform speed of about fifteen miles an hour, the French railway system being such that though progress is comparatively slow there are no waits or delays, no train being held up for the passing of a faster train.

The journey is a busy one, particulars of all patients are recorded, including the numbers of the bunks occupied. Wounds have to be re-dressed, occasionally an operation must be carried out; the patients have to be fed; sometimes two or three meals are required, depending upon the length of the journey, in addition to the administration of beef-tea and other stimulants. As a train will sometimes carry five hundred cases, the number varying according to the proportion of sitting and lying cases, as well as its own *personnel*, the medical officer in charge has varied preoccupations. He has to cater for a large establishment, constantly changing in extent, in addition to the strictly professional side of his duties. But the R.A.M.C. has proved one thing in this war quite clearly: that a medical man can, when he is put to it, capably exercise more functions than those of a healer.

The trains arrive at the base. If there are two sidings much delay is saved, and the ambulances may more rapidly be despatched to the hospitals.

### Ambulance Work.

The average time taken in emptying an ambulance train is fifty minutes if the proportions of sitting and lying cases are normal. If the cases are all sitting, a train can be cleared in twenty minutes. It is pathetic to see the care with which the wounded soldiers look after their few belongings, especially when these include trophies. A stretcher will be lifted out bearing a man with his head swathed in bandages, and on his chest will lie a German helmet; this souvenir will accompany the patient until he has, in hospital language, been "treated to a conclusion."

A word may be said here as to motor-ambulances. Most of them are of admirable construction, but others, especially those of the Canadian Red Cross "Buwick" type, are unnecessarily high in the roof, with too much space between the lower and upper stretcher on either side. This condition makes the car heavier, and causes extra strain to the bearers in dealing with the upper stretchers. Yet the space between is not sufficient to accommodate two rows of sitting patients under the upper tiers, which, were this possible, could be utilised for two lying cases in addition; perhaps such a use would be a justification of this feature in construction. The better types have just enough room for the four stretchers, with a little seat for a less helpless man to sit with his back to the driver, and, when the upper tiers are removed, comfortably take the usual complement of sitters.

The base hospitals are called "general" and "stationary"; the latter are usually smaller than the former, but there appears, for all practical purposes at present, to be no difference in their work. It may be explained that originally, under the Army organisation, paradoxical though it sounds, a stationary hospital was one that could be moved about according to the positions of the armies, being, as a rule, placed somewhere between the bases and the clearing stations. At the base will also generally be found Canadian, Australian, Red Cross, and other voluntary hospitals, which, for descriptive purposes, may be classed as "general." Amongst organisations serving hospitals at the base will be found laundries, bakeries, medical and other stores, grave-registration bureaux, and depôts for dealing with kitchen and general waste.

*(To be continued.)*

